# Bilobalide inhibits inflammation and promotes the expression of Aβ degrading enzymes in astrocytes to rescue neuronal deficiency in AD models

**DOI:** 10.1038/s41398-021-01594-2

**Published:** 2021-10-20

**Authors:** Jun Xiang, Feng Yang, Wen Zhu, Min Cai, Xiang-Ting Li, Jing-Si Zhang, Zhong-Hai Yu, Wen Zhang, Ding-Fang Cai

**Affiliations:** 1 Department of Integrative Medicine, Zhongshan Hospital, Fudan University, 200032 Shanghai, China; 2 Laboratory of Neurology, Institute of Integrative Medicine, Fudan University, 200032 Shanghai, China; 3 Department of Neurology, Shuguang hospital affiliated to Shanghai University of TCM, 201203 Shanghai, China; 4 Department of Traditional Chinese Medicine, the Sixth People’s Hospital Affiliated to Shanghai Jiao Tong University, 200233 Shanghai, China

**Keywords:** Diseases, Molecular neuroscience

## Abstract

The pathogenesis of Alzheimer’s disease (AD) involves multiple cell types including endothelial cells, glia, and neurons. It suggests that therapy against single target in single cell type may not be sufficient to treat AD and therapies with protective effects in multiple cell types may be more effective. Here, we comprehensively investigated the effects of bilobalide on neuroinflammation and Aβ degrading enzymes in AD cell model and mouse model. We find that bilobalide inhibits Aβ-induced and STAT3-dependent expression of TNF-α, IL-1β, and IL-6 in primary astrocyte culture. Bilobalide also induces robust expression of Aβ degrading enzymes like NEP, IDE, and MMP2 to facilitate astrocyte-mediated Aβ clearance. Moreover, bilobalide treatment of astrocyte rescues neuronal deficiency in co-cultured APP/PS1 neurons. Most importantly, bilobalide reduces amyloid and inflammation in AD mouse brain. Taken together, the protective effects of bilobalide in in vitro cultures were fully recapitulated in in vivo AD mouse model. Our study supports that bilobalide has therapeutic potential for AD treatment.

## Introduction

Alzheimer’s disease (AD) is a devastating neurodegenerative disorder characterized by progressive deterioration in cognition and daily function [1]. The key pathological changes observed in AD brain tissue are amyloid-β (Aβ) peptide deposited as extracellular plaques and hyperphosphorylated tau (p-tau) accumulated as intracellular neurofibrillary tangles (NFTs). Additional changes include reactive gliosis and widespread loss of neurons and synapses. AD is a complex and progressive neurodegenerative disorder, and multiple cell types were involved in its pathogenesis including endothelial cells, glia and neurons [2, 3]. Unfortunately, nearly all clinical trials in AD patients have failed and it suggests that therapies against single target in single cell type may not be sufficient to treat AD. Thus, therapies with protective effects in multiple cell types may be more effective.

Bilobalide (BB) [4] is a sesquiterpene trilactone constituent that accounts for ~2.9% of the standardized Ginkgo biloba extract EGb761®, which has been used as phytomedicine or food supplement in many countries [5, 6]. Interestingly, Ginkgo biloba extract seems to show protective effects for dementia according to numerous epidemiological studies [7]. However, its underlying mechanism is far from clear and remains to be explored. Importantly, studies have shown that bilobalide could promote neurogenesis and synaptogenesis in hippocampal neurons [8], and prevent Amyloid-β-induced depolarization of cortical neurons [9]. In addition, bilobalide shows antineuroinflammatory effects in LPS-activated primary microglial cells [10]. Thus, it seems that bilobalide has protective effects in multiple cell types, but it’s unclear whether and how could bilobalide modulate astrocytes in AD.

In this study, we report that bilobalide acts on astrocytes to inhibit STAT3-depednet expression of inflammation cytokines and promotes the expression of Aβ degrading enzymes to rescue neuronal deficiency in in vitro and in vivo AD models. Thus, bilobalide may be a potential AD intervention with protective effects in multiple cell types involved AD pathogenesis.

## Methods

### Animals

Our study was approved by the Institutional Ethics Committee of Zhongshan Hospital, Fudan University. All animal experiments were performed in accordance with the Institutional Animal Care and Use Committee. APP/PS1 mice were from Jackson laboratory (#004462). Male APP/PS1 mice (12 month old) were intraperitoneally injected with bilobalide (*n* = 6, 0.5 mg/kg) or DMSO control (*n* = 6) every week for 4 weeks.

### Chemical reagents

Aβ42 peptide was from American Peptide. Bilobalide was from Sigma (B9031). STAT3 inhibitor C188-9 was from Selleckchem (S8605). All other chemical reagents were from Sigma.

### Cell cultures

The primary astrocyte cultures were obtained from P1 pups of C57BL/6 mouse. Briefly, mice were decapitated and brain was dissected to obtain the cortex. Cortex tissues were minced with cold phosphate buffered saline, digested with 0.25% trypsin at 37 °C for 6 min, resuspended as a single cell suspension in culture medium including DMEM/F-12 medium (Gibco, Gaithersburg, MD, USA) and 10% fetal bovine serum (FBS; Gibco, Gaithersburg, MD, USA) and seeded into culture flask precoated with Poly-D-lysine. Cells were maintained at 37 °C in a 95% humid atmosphere with 5% CO_2_. Microglia and other nonadherent cells were removed by gently shaking the culture flasks at 200 rpm for 2 h. Astrocyte cultures from day in vitro (DIV) 7 to DIV10 were detached with 0.25% trypsin +0.02% EDTA in PBS and plated for downstream experiments. The purity of the astrocyte culture is above 95%. Primary cortical neurons were cultured from P0 pups of APP/PS1 or C57BL/6 mice and cultured in neurobasal supplemented with B27 and 0.5 mM Glutamax for 14 days.

### Co-cultures

The co-culture system consists of lower and upper chambers, which were separated by a selectively permeable membrane with 0.3 μm-diameter pores (Corning, Transwell 3450). The primary mouse astrocytes were plated in the upper chamber. Primary neurons were plated in the lower chamber and were co-cultured with astrocytes to simulate the microenvironment of central nervous system.

### ELISA

Aβ ELISA was performed as previously described [11]. In brief, mouse forebrains were homogenized in TBST and centrifuged. The supernatant was collected for TBST soluble Aβ, and the pellet was further homogenized in GuHCl for TBST insoluble Aβ. Aβ levels in the mouse forebrain and culture medium were quantified by Amyloid beta 40 Human ELISA Kit (Invitrogen, KHB3482) and Amyloid beta 42 Human ELISA Kit (Invitrogen, KHB3544). TNF-α, IL-1β, and IL-6 levels in mouse forebrain and culture medium were quantified by Mouse TNF alpha ELISA Kit (ab208348, abcam), Mouse IL-1 beta ELISA Kit (ab197742, abcam) and Mouse IL-6 ELISA Kit (ab222503, abcam).

### Western blot

For cultured cells and culture medium, proteins were extracted using sodium dodecyl sulfate lysis buffer (2% sodium dodecyl sulfate, 10% glycerol, 0.1 mM dithiothreitol, and 0.2 M Tris–HCl, pH 6.8). For brain tissues, proteins were extracted using RIPA buffer. Protein samples (50ug) were resolved by SDS-PAGE and detected with following antibodies: STAT3 (9139, CST), Phospho-STAT3 (Tyr705) (9145, CST), IDE (ab32216, abcam), MMP2 (87809, CST), NEP (ab255609, abcam), PSD-95 (3450, CST), Synapsin-1 (5297, CST), GluR1 (AB1504, Milllipore), and Synaptophysin (36406, CST). The protein bands were quantified by densitometry analysis using Image J software and the intensity of each target protein was normalized by **α–**Tubulin intensity.

### Quantitative real-time PCR

Total RNA (1 μg) was reverse-transcribed into cDNA with random-hexamer primer mix using M-MLV Reverse Transcriptase (Promega) according to the manufacturer’s instructions. Quantitative real-time PCR (qRT-PCR) was performed with TaqMan assays for IDE (Mm00473077_m1), MMP2 (Mm00439498_m1), NEP (Mm01285046_g1), TNF-α (Mm00443258_m1), IL-1β (Mm00434228_m1), and IL-6 (Mm00446190_m1).

### Aβ clearance assay

Aβ clearance assay was performed with minor modifications [12]. Astrocytes were treated with bilobalide and then switched to serum-free DMEM with 1 ug/ml dissolved Aβ42 and 10 ug /ml BSA for 12 h. After incubation, the culture medium was collected and Aβ42 level was measured by ELISA.

### CCK-8 assay and ROS assay

The cell viability and cell proliferation were assessed by CCK-8 assay as previously described [13]. Cellular ROS level was measured by Reactive Oxygen Species Assay Kit (Beyotime Institute of Biotechnology, Jiangsu, China) as previously described [13].

### STAT3 luciferase assay

To monitor the transcriptional activity of STAT3, STAT3 reporter lentivirus (PLV-10065, Cellomics Technology), which expresses Firefly luciferase under the control of a minimal CMV promoter and tandem repeats of STAT3 transcriptional response element was used to infect astrocytes at MOI of 10 for 72 h before Aβ42 treatment. Then, the astrocyte lyaste was analyzed by luciferase assay.

### Statistical analysis

Statistical analysis was performed using GraphPad Prism software. All data were presented as mean ± SD and statistical analysis was performed by two-tailed Student *t*-test for two groups and one way ANOVA with Newman-Keuls post-hoc test for more than two groups. Statistically significant differences were defined as *P* < 0.05. For all, *P < 0.05, **P < 0.01, ***P < 0.001.

## Results

### Aβ42 induces STAT3-dependent inflammation responses in primary astrocyte culture

**Fig. 1:**
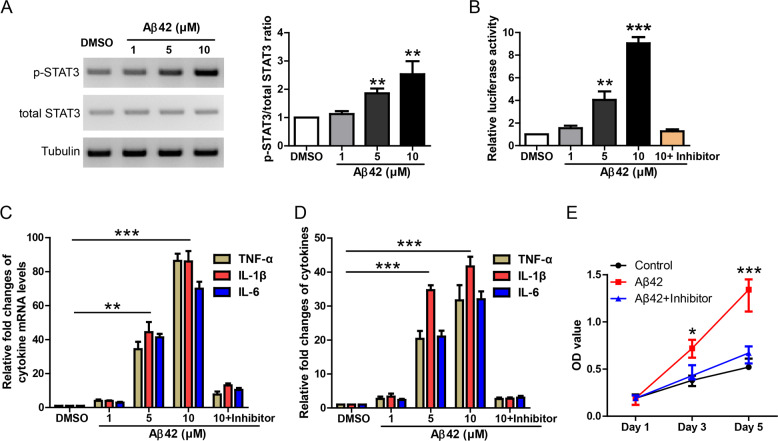
Aβ42 induces STAT3-dependent inflammation responses in primary astrocyte culture. **A** Western blot and its quantification showing STAT3 protein levels in astrocyte culture treated by Aβ42 at indicated concentrations. **B** STAT3 luciferase reporter assay showing STAT3 transcription activity in astrocyte culture treated by Aβ42 at indicated concentrations and STAT3 inhibitor. **C** Quantitative real-time PCR results showing the levels of TNF-α, IL-1β, and IL-6 in the astrocyte culture treated by Aβ42 at indicated concentrations and STAT3 inhibitor. **D** ELISA results showing the levels of TNF-α, IL-1β, and IL-6 in the astrocyte supernatant under the treatment of Aβ42 at indicated concentrations and STAT3 inhibitor. **E** CCK-8 assay showing the astrocyte proliferation under the treatment of Aβ42 at indicated concentrations and STAT3 inhibitor. *P < 0.05, **P < 0.01, ***P < 0.001.

Recent studies have shown that STAT3 is essential for astrocyte activation in AD mouse brain [14, 15]. Hence, we first tried to determine whether Aβ42 treatment could induce STAT3-dependent inflammation response in primary astrocyte culture. Astrocytes were incubated with Aβ42 at indicated concentrations (1, 5, 10 μM) for 24 h and then the astrocyte lyaste was analyzed by western blot. WB result showed that Aβ42 increased p-STAT3 in a dose-dependent manner (Fig. 1A). In addition, we used a lentiviral luciferase reporter, which contains STAT3 response element to further confirm Aβ42-induced STAT3 activation. Astrocytes infected with lentiviral STAT3 luciferase reporter were incubated with Aβ42 and then the astrocyte lyaste was analyzed by luciferase assay (Fig. 1B). The result showed that Aβ42 increased luciferase activity in a dose-dependent manner and this was blocked by a potent STAT3 inhibitor C188-9 (final concentration 5 μM). Taken together, these data suggest that Aβ42 treatment activates STAT3 signaling in primary astrocyte culture.

As numerous inflammation cytokines are target genes of STAT3 signaling, Aβ42-induced inflammation response was measured by qRT-PCR in the astrocyte lyaste and ELISA in the culture medium. Indeed, qRT-PCR (Fig. 1C) and ELISA (Fig. 1D) results showed that inflammation cytokines like TNF-α, IL-1β, and IL-6 were dramatically induced by Aβ42 in a dose-dependent manner but inhibited largely by STAT3 inhibitor C188-9. In addition, CCK-8 assay showed that Aβ42 treatment promoted astrocyte proliferation, which was blocked by C188-9 (Fig. 1E). Taken together, these results suggest that Aβ42 induces STAT3-dependent inflammation responses in primary astrocyte culture.

### Bilobalide inhibits STAT3-dependent inflammation responses in Aβ42-treated astrocyte culture

**Fig. 2:**
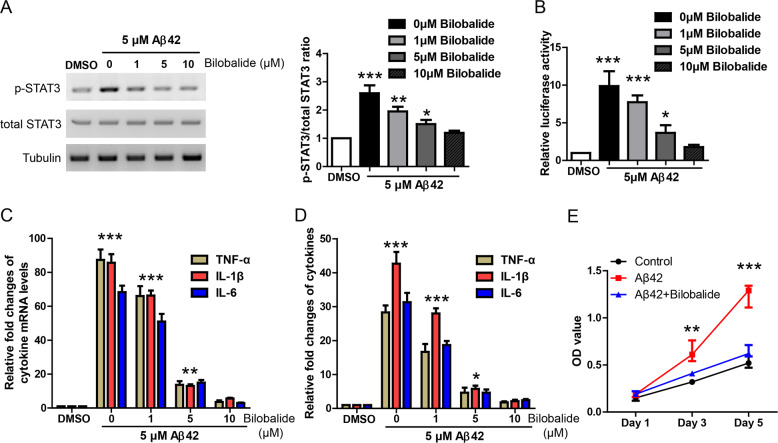
Bilobalide inhibits STAT3-dependent inflammation responses in Aβ42-treated astrocyte culture. **A** Western blot and its quantification showing STAT3 protein levels in astrocyte culture treated by 5 μM Aβ42 and bilobalide at indicated concentrations. **B** STAT3 luciferase reporter assay showing STAT3 transcription activity in astrocyte culture treated by 5 μM Aβ42 and bilobalide at indicated concentrations. **C** Quantitative real-time PCR results showing the levels of TNF-α, IL-1β, and IL-6 in the astrocyte culture treated by 5 μM Aβ42 and bilobalide at indicated concentrations. **D** ELISA results showing the levels of TNF-α, IL-1β, and IL-6 in the astrocyte supernatant under the treatment of 5 μM Aβ42 and bilobalide at indicated concentrations. **E** CCK-8 assay showing the astrocyte proliferation under the treatment of 5 μM Aβ42 and bilobalide at indicated concentrations. *P < 0.05, **P < 0.01, ***P < 0.001.

To investigate the potential effects of bilobalide, astrocytes were incubated with Aβ42 alone (5 μM) or together with bilobalide at indicated concentrations (1, 5, 10 μM) for 24 h. WB result of astrocyte lyaste showed that Aβ42 activated p-STAT3 level while bilobalide inhibited p-STAT3 dose dependently (Fig. 2A). Similarly, Aβ42 increased luciferase activity in STAT3 luciferase reporter assay while bilobalide inhibited STAT3 luciferase activity dose dependently (Fig. 2B).

More importantly, qRT-PCR in astrocyte lyaste (Fig. 2C) and ELISA in the culture medium (Fig. 2D) showed that Aβ42-induced TNF-α, IL-1β, and IL-6 were greatly inhibited by bilobalide in a dose-dependent way. In addition, CCK-8 assay showed that bilobalide inhibited Aβ42-induced astrocyte proliferation (Fig. 2E). Taken together, these results suggest that bilobalide inhibits STAT3-dependent inflammation responses in Aβ42-treated astrocyte culture.

### Bilobalide induces the expression of Aβ degrading enzymes to facilitate Aβ clearance in primary astrocyte culture

**Fig. 3:**
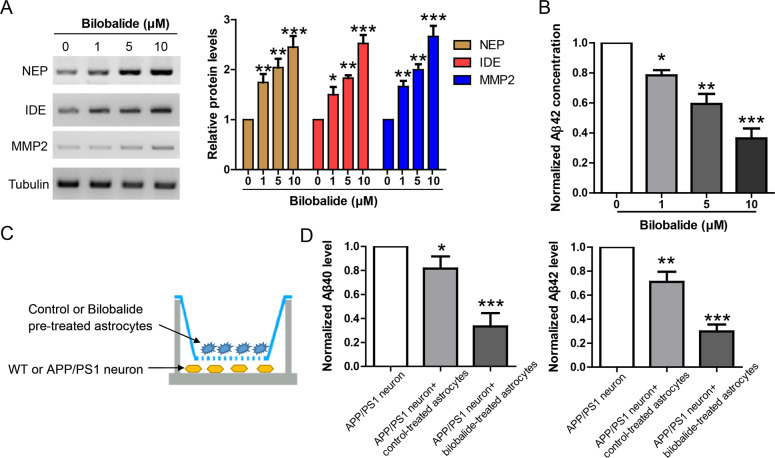
Bilobalide induces the expression of Aβ degrading enzymes to facilitate Aβ clearance in primary astrocyte culture. **A** Western blot and its quantification showing the protein levels of NEP, IDE, and MMP2 in astrocyte culture treated by bilobalide at indicated concentrations. **B** Aβ clearance assay showing Aβ42 levels in the culture medium of astrocytes treated by bilobalide at indicated concentrations. **C** Diagram showing the astrocyte-neuron co-culture in transwell system. **D** ELISA results showing Aβ40 and Aβ42 levels in the culture medium of APP/PS1 neuron co-cultured with control or bilobalide (5 μM) pretreated astrocytes. *P < 0.05, **P < 0.01, ***P < 0.001.

It’s well established that astrocytes express high levels of Aβ degrading enzymes like NEP [16], IDE [17] and MMP2 [18] to maintain Aβ homeostasis in the brain. To investigate whether bilobalide could affect astrocyte-mediated Aβ42 clearance, we incubated astrocytes with bilobalide at indicated concentrations (1, 5, 10 μM) for 24 h. The astrocyte lyaste was analyzed by western blot for NEP, IDE, and MMP2, and the result showed that bilobalide upregulated NEP, IDE, and MMP2 (Fig. 3A). It suggests that bilobalide might enhance astrocyte-mediated Aβ clearance. To confirm this, we used an Aβ clearance assay in which astrocytes were incubated with Aβ42 (1 μg/ml). As astrocytes uptake Aβ42 from the culture medium and degrade these Aβ42 via enzymes like NEP, IDE, and MMP2, the Aβ42 level in the culture medium would be reduced time dependently. We first treated astrocytes with bilobalide at indicated concentrations for 24 h, and then the culture medium was replaced by medium containing 1 μg/ml Aβ42. After 12 h of incubation, the Aβ42 level in the culture medium was measured by ELISA. The result showed bilobalide reduced Aβ42 level in the culture medium dose dependently (Fig. 3B).

To fully recapitulate the in vivo interaction between ascrocytes and neurons in AD brain, we established a transwell based co-culture system (Fig. 3C). The primary cortical neurons from APP/PS1 mouse were plated in the lower chamber while the astrocytes were plated in up chamber and pretreated with bilobalide before its co-culture with APP/PS1 neurons. ELISA result showed that endogenous Aβ40 and Aβ42 levels in neuronal culture medium were reduced by co-culture with control-treated ascrocytes. Co-culture with bilobalide-treated ascrocytes further facilitated this Aβ clearance (Fig. 3D). Taken together, these results suggest that bilobalide could induce the expression of Aβ degrading enzymes to facilitate astrocyte-mediated Aβ clearance.

### Bilobalide rescues neuronal deficiency in astrocyte-APP/PS1 neuron co-cultures

**Fig. 4:**
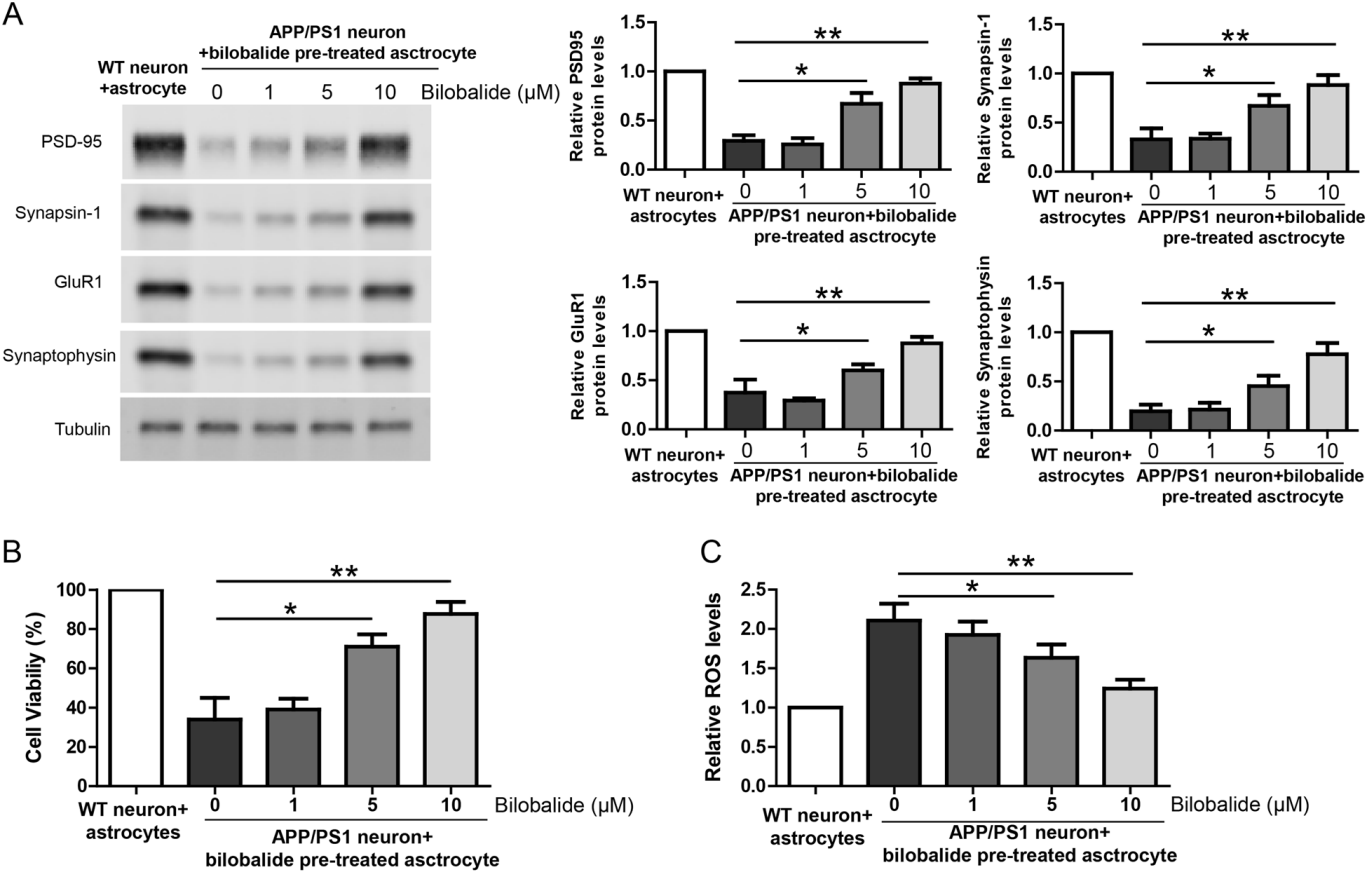
Bilobalide rescues neuronal deficiency in astrocyte-APP/PS1 neuron co-cultures. **A** Western blot and its quantification showing the protein levels of PSD-95, Synapsin-1, GluR1, and Synaptophysin in the lysate of wild-type neuron or APP/PS1 neuron co-cultured with astrocyte pretreated with Bilobalide. **B** CCK-8 assay showing neuronal viability of wild-type neuron or APP/PS1 neuron co-cultured with astrocyte pretreated with Bilobalide. **C** ROS levels in the lysate of wild-type neuron or APP/PS1 neuron co-cultured with astrocyte pretreated with Bilobalide. *P < 0.05, **P < 0.01.

In the same co-culture of astrocyte-APP/PS1 neurons, the neuronal lysate was analyzed by western blot for synaptic proteins including PSD-95, Synapsin-1, GluR1, and Synaptophysin. The results showed that the expression of synaptic proteins was impaired in APP/PS1 neurons compared to WT neurons while bilobalide pretreated asctrocyte rescued the expression of synaptic proteins in APP/PS1 neurons (Fig. 4A). In addition, cell viability and ROS production in neurons were also measured by CCK-8 assay and DCFH-DA probe, respectively. The results showed that APP/PS1 neurons had lower cell viability compared to WT neurons and bilobalide pretreated asctrocyte improved cell viability of APP/PS1 neurons (Fig. 4B). Similarly, APP/PS1 neurons had much higher ROS levels compared to WT neurons and bilobalide pretreated asctrocyte greatly inhibited ROS levels in APP/PS1 neurons (Fig. 4C). Taken together, these results suggest that Bilobalide is protective in astrocyte-APP/PS1 neuron co-cultures.

### Bilobalide shows better protective effects in co-cultures compared to neuron culture

**Fig. 5:**
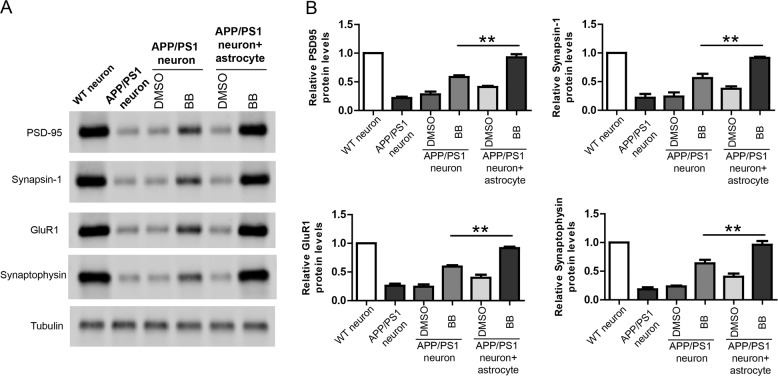
Bilobalide shows better protective effects in co-cultures compared to neuron culture. **A** Western blot and **B** its quantification showing the protein levels of PSD-95, Synapsin-1, GluR1, and Synaptophysin in the lysate of wild-type neuron or APP/PS1 neuron co-cultured with astrocyte pretreated with Bilobalide. **P < 0.01.

We also compared the rescue effects of BB in APP neuron culture or in APP neuron-astrocyte co-culture (Fig. 5). WB results show that in APP neuron culture, BB treatment restored synaptic proteins to 50% of WT neuron culture. Under the same conditions, BB treatment restored synaptic proteins in APP neuron-astrocyte co-culture to 90% of WT neuron culture. These results strongly suggest that BB could achieve better neuroprotective effects via acting on multiple cell types.

### Bilobalide reduces amyloid and inflammation in AD mouse brain

**Fig. 6:**
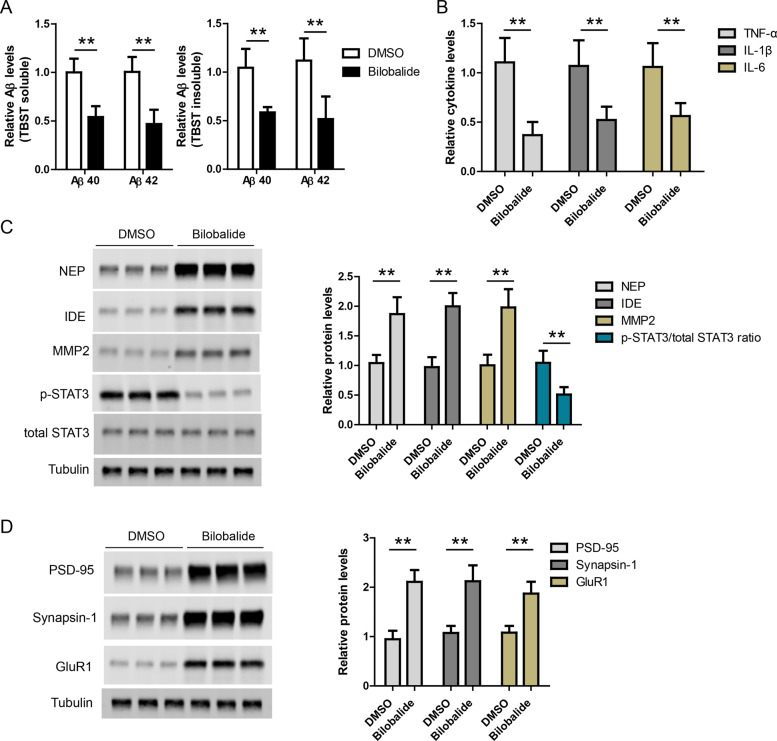
Bilobalide reduces amyloid and inflammation in AD mouse brain. **A** ELISA results showing Aβ42 and Aβ40 levels in the brain lysates of APP/PS1 mice treated with DMSO (*n* = 6) or Bilobalide (*n* = 6). **B** ELISA results showing TNF-α, IL-1β, and IL-6 levels in the brain lysates of APP/PS1 mice treated with DMSO (*n* = 6) or Bilobalide (*n* = 6). **C** Western blot and its quantification showing the protein levels of NEP, IDE, MMP2, and STAT3 in the brain lysates of APP/PS1 mice treated with DMSO (*n* = 6) or Bilobalide (*n* = 6). **D** Western blot and its quantification showing the protein levels of PSD-95, Synapsin-1, and GluR1 in the brain lysates of APP/PS1 mice treated with DMSO (*n* = 6) or Bilobalide (*n* = 6). **P < 0.01.

At last, we evaluated the effects of bilobalide in APP/PS1 mouse brain. Bilobalide (*n* = 6) or DMSO control (*n* = 6) was administrated in APP/PS1 mice by intraperitoneal injection. First, Aβ40 and Aβ42 levels in brain lysates were measured by ELISA and the result showed that Aβ levels were lower in bilobalide group compared to DMSO group (Fig. 6A). Then, TNF-α, IL-1β, and IL-6 levels in brain lysates were measured by ELISA. The results showed that TNF-α, IL-1β, and IL-6 levels were lower in Bilobalide group compared to DMSO group (Fig. 6B). The protein levels of STAT3, NEP, IDE, and MMP2 in brain lysates were also measured by western blot. The results showed that NEP, IDE, and MMP2 levels were higher while p-STAT3 level was lower in bilobalide group compared to DMSO group (Fig. 6C). At last, bilobalide treatment also increased synaptic proteins including PSD-95, Synapsin-1, and GluR1 in AD mouse brain (Fig. 6D).

Taken together, the protective effects of bilobalide in in vitro cultures were fully recapitulated in in vivo AD mouse model and these results suggest that bilobalide has therapeutic potential in AD models.

## Discussion

In this study, we comprehensively investigated the effects of bilobalide in in vitro and in vivo AD models. We find that bilobalide inhibits Aβ-induced and STAT3-dependent expression of TNF-α, IL-1β, and IL-6 in primary astrocyte culture. Bilobalide also induces robust expression of Aβ degrading enzymes like NEP, IDE, and MMP2 to facilitate astrocyte-mediated Aβ clearance. Moreover, bilobalide treatment of astrocyte rescues neuronal deficiency in co-cultured APP/PS1 neurons. Most importantly, bilobalide reduces amyloid and inflammation in AD mouse brain. Taken together, these results suggest that bilobalide has therapeutic potential in AD mouse model.

Several compounds have been developed to target Aβ, but they were not successful in clinical trials to treat AD patients [19]. Although different reasons may have contributed to the failures, it’s important to note that multiple cell types were involved in the pathogenesis of AD and current drugs usually focus on single target in single cell type [20]. Besides the detrimental loss of neurons and synapses, astrocytes, and microglia were overactivated and long-term uncontrolled inflammation could amplify brain damages [21]. In addition, vascular endothelial cells were also damaged in AD, leading to abnormal blood supply and compromised permeability of blood–brain barrier and infiltration of peripheral immune cells [22]. Thus, drugs against single target in single cell type may not be sufficient to modify the underlying pathogenesis involving multiple cell types. Therefore, it’s of great interest to explore drugs with broad protective effects in multiple cell types.

Bilobalide is an interesting compound as it shows broad effects in various cell types. Studies have shown that bilobalide could promote neurogenesis and synaptogenesis in hippocampal neurons, prevent Amyloid-β-induced depolarization of cortical neurons and reduce apoptosis in SH-SY5Y cells. In addition, bilobalide showed antineuroinflammatory effects in LPS-activated primary microglial cells. Bilobalide also protects endothelial cells from hypoxia-induced ATP decrease [23]. Here, we further show that bilobalide acts on astocytes to rescue AD phenotypes. Taken together, our and other independent studies support that bilobalide might be an effective treatment with multiple protective effects for AD patients.

One important finding in our study is that BB could induce NEP expression in astrocytes and AD mouse brain. However, we notice that there is a study suggesting that BB is unable to change NEP expression [24]. It’s likely the differences in the experimental conditions could explain the discrepancy. First, in our study, BB was intraperitoneally injected in 12-month APP/PS1 mice. However, EGb761 was supplemented in the diet for 6-month WT mice in the above reference. Second, we measured NEP protein levels while NEP mRNA levels were measured in the above reference. It’s possible that BB upregulated NEP expression via post-transcriptional mechanism. At last, although not significant, EGb761 did show a clear trend of upregulation in NEP mRNA levels after EGb761 intervention in both hippocampus and cortex in the above reference.

## Conclusions

In conclusion, we provide evidence that bilobalide acts on astrocytes to inhibit STAT3-depednet expression of inflammation cytokines and promote the expression of Aβ degrading enzymes to rescue neuronal deficiency in in vitro and in vivo AD models.

## Data Availability

All data generated or analyzed during this study are included in this published article.

## References

[1] Hardy J, Selkoe DJ (2002). The amyloid hypothesis of Alzheimer’s disease: progress and problems on the road to therapeutics. Science..

[2] Zlokovic BV (2011). Neurovascular pathways to neurodegeneration in Alzheimer’s disease and other disorders. Nat Rev Neurosci.

[3] Iadecola C (2017). The neurovascular unit coming of age: a journey through neurovascular coupling in health and disease. Neuron..

[4] van Beek TA, Montoro P (2009). Chemical analysis and quality control of Ginkgo biloba leaves, extracts, and phytopharmaceuticals. J Chromatogr A..

[5] Wagner H, Ulrich-Merzenich G. Evidence and Rational Based Research on Chinese Drugs. Springer-Verlag Wien, 471–87.

[6] Jiratchariyakul W, Mahady GB. Overview of Botanical Status in EU, USA, and Thailand. Evidence-Based Complementary and Alternative Medicine. 2013;2013:480128. 10.1155/2013/480128. 10.1155/2013/480128 PMC3818839 24228061

[7] Yuan QJ, Wang CW, Shi J, Lin ZX (2017). Effects of Ginkgo biloba on dementia: an overview of systematic reviews. J Ethnopharmacol..

[8] Tchantchou F, Lacor PN, Cao Z, Lao L, Hou Y, Cui C (2009). Stimulation of neurogenesis and synaptogenesis by bilobalide and quercetin via common final pathway in hippocampal neurons. J Alzheimers Dis.

[9] Kuo LC, Song YQ, Yao CA, Cheng IH, Chien CT, Lee GC (2019). Ginkgolide A prevents the amyloid-beta-induced depolarization of cortical neurons. J Agr Food Chem..

[10] Gargouri B, Carstensen J, Bhatia HS, Huell M, Dietz G, Fiebich BL (2018). Anti-neuroinflammatory effects of Ginkgo biloba extract EGb761 in LPS-activated primary microglial cells. Phytomedicine..

[11] Lu R, Wang J, Tao R, Wang J, Zhu T, Guo W (2018). Reduced TRPC6 mRNA levels in the blood cells of patients with Alzheimer’s disease and mild cognitive impairment. Mol Psychiatr..

[12] Wang J, Lu R, Yang J, Li H, He Z, Jing N, et al. TRPC6 specifically interacts with APP to inhibit its cleavage by gamma-secretase and reduce A beta production. Nat Commun. 2015;6. 10.1038/ncomms9876 PMC4696454 26581893

[13] Xiang J, Zhu W, Yang F, et al. Melatonin-induced ApoE expression in mouse astrocytes protects endothelial cells from OGD-R induced injuries. Transl Psychiatry. 2020;10:181. 10.1038/s41398-020-00864-9 PMC7280243 32513932

[14] Reichenbach N, Delekate A, Plescher M, Schmitt F, Krauss S, Blank N, et al. Inhibition of Stat3-mediated astrogliosis ameliorates pathology in an Alzheimer’s disease model. EMBO Mol Med. 2019;11. 10.15252/emmm.201809665 PMC6365929 30617153

[15] Ben Haim L, Ceyzériat K, Carrillo-de Sauvage MA, Aubry F, Auregan G, Guillermier M (2015). The JAK/STAT3 pathway is a common inducer of astrocyte reactivity in Alzheimer’s and Huntington’s diseases. J Neurosci..

[16] Yamamoto N, Arima H, Naruse K, Kasahara R, Taniura H, Hirate H (2013). Ketamine reduces amyloid beta-protein degradation by suppressing neprilysin expression in primary cultured astrocytes. Neurosci Lett..

[17] Son SM, Cha MY, Choi H, Kang S, Choi H, Lee MS (2016). Insulin-degrading enzyme secretion from astrocytes is mediated by an autophagy-based unconventional secretory pathway in Alzheimer disease. Autophagy..

[18] Yin KJ, Cirrito JR, Yan P, Hu X, Xiao Q, Pan X (2006). Matrix metalloproteinases expressed by astrocytes mediate extracellular amyloid-beta peptide catabolism. J Neurosci..

[19] Cummings J (2018). Lessons learned from Alzheimer disease: clinical trials with negative outcomes. Clin Transl Sci..

[20] Gong CX, Liu F, Iqbal K (2018). Multifactorial hypothesis and multi-targets for Alzheimer’s disease. J Alzheimers Dis..

[21] Skaper SD, Facci L, Zusso M, Giusti P. An inflammation-centric view of neurological disease: beyond the neuron. Front Cell Neurosci. 2018;12. 10.3389/fncel.2018.00072 PMC5871676 29618972

[22] Sweeney MD, Sagare AP, Zlokovic BV (2018). Blood-brain barrier breakdown in Alzheimer disease and other neurodegenerative disorders. Nat Rev Neurol.

[23] Janssens D, Michiels C, Delaive E, Eliaers F, Drieu K, Remacle J (1995). Protection of hypoxia-induced Atp decrease in endothelial-cells by Ginkgo-biloba extract and bilobalide. Biochem Pharmacol..

[24] Augustin S, Rimbach G, Augustin K, Cermak R, Wolffram S (2009). Gene regulatory effects of Ginkgo biloba extract and its flavonol and terpenelactone fractions in mouse brain. J Clin Biochem Nutr..

